# Impact of acute psychosocial stress on attentional control in humans. A study of evoked potentials and pupillary response

**DOI:** 10.1016/j.ynstr.2023.100551

**Published:** 2023-06-12

**Authors:** F. Rojas-Thomas, C. Artigas, G. Wainstein, Juan-Pablo Morales, M. Arriagada, D. Soto, A. Dagnino-Subiabre, J. Silva, V. Lopez

**Affiliations:** a Laboratorio de Psicología Experimental y Neurociencias, Escuela de Psicología, Pontificia Universidad Católica de Chile, Santiago, Chile; b Departamento de Biología, Universidad Autónoma de Chile, Santiago, Chile; c Programa de Doctorado en Neurociencia, Centro Interdisciplinario en Neurociencia, Pontificia Universidad Católica de Chile, Santiago, Chile; d Departamento de Psiquiatría, Escuela de Medicina y Centro Interdisciplinario de Neurociencia, Pontificia Universidad Católica de Chile, Santiago, Chile; e Laboratorio de Neurobiología del Estrés, Instituto de Fisiología, CENFI, Facultad de Ciencias, Universidad de Valparaíso, Valparaíso, Chile; f Center for Social and Cognitive Neuroscience (CSCN), School of Psychology, Universidad Adolfo Ibáñez, Santiago de Chile, Chile; g College of Veterinary Medicine, Faculty of Medical Sciences, Bernardo O'Higgins University, Santiago, Chile; h Instituto de Bienestar Socioemocional (IBEM), Facultad de Psicología, Universidad del Desarrollo, Santiago, Chile; i Facultad de Educación Psicología y Familia, Universidad Finis Terrae, Santiago, Chile

**Keywords:** Attention, EEG, Acute psychosocial stress, Pupil diameter, Locus coeruleus, Noradrenergic system

## Abstract

Psychosocial stress has increased considerably in our modern lifestyle, affecting global mental health. Deficits in attentional control are cardinal features of stress disorders and pathological anxiety. Studies suggest that changes in the locus coeruleus-norepinephrine system could underlie the effects of stress on top-down attentional control. However, the impact of psychosocial stress on attentional processes and its underlying neural mechanisms are poorly understood. This study aims to investigate the effect of psychosocial stress on attentional processing and brain signatures. Evoked potentials and pupillary activity related to the oddball auditory paradigm were recorded before and after applying the Montreal Imaging Stress Task (MIST). Electrocardiogram (ECG), salivary cortisol, and subjective anxiety/stress levels were measured at different experimental periods. The control group experienced the same physical and cognitive effort but without the psychosocial stress component. The results showed that stressed subjects exhibited decreased P3a and P3b amplitude, pupil phasic response, and correct responses. On the other hand, they displayed an increase in Mismatch Negativity (MMN). N1 amplitude after MIST only decreased in the control group. We found that differences in P3b amplitude between the first and second oddball were significantly correlated with pupillary dilation and salivary cortisol levels. Our results suggest that under social-evaluative threat, basal activity of the coeruleus-norepinephrine system increases, enhancing alertness and decreasing voluntary attentional resources for the cognitive task. These findings contribute to understanding the neurobiological basis of attentional changes in pathologies associated with chronic psychosocial stress.

## 1 Introduction

As posited by the theory of attentional competition, the discernment of relevant information from our surroundings is governed by two distinct attentional mechanisms (Desimone and Duncan, 1995; Katsuki and Constantinidis, 2014). Reflexive attention is a stimulus-driven process (Bottom-up) in which a sensory event, for example, a roar, movement, or possible threats, captures our attention (Egeth and Yantis, 1997; Sänger et al., 2014). Voluntary attention is a goal-driven process (Top-Down), meaning that our goals, expectations, and rewards guide what we attend (Desimone and Duncan, 1995). The balance between Bottom-up and Top-Down attentional bias allows us to respond appropriately to the diversity of the environment. Clinical studies propose that attentional mechanisms are altered in some mood disorders (Beevers et al., 2015; Drueke et al., 2020). For example, attentional bias toward threat-related or negative information is a critical cognitive marker in stress disorders and pathological anxiety (Bar-Haim et al., 2007; Peckham et al., 2010). In this line, chronic exposure to stress can generate structural and functional changes in critical structures related to detecting threats (Jedema et al., 2001; Li et al., 2018; Vyas et al., 2003). These alterations could underlie the abnormal attentional and excessive threat perception associated with stress disorders (Chattarji et al., 2015; Morris et al., 2020; Naegeli et al., 2018). However, the attentional mechanisms related to normal stress response and its link with a mood disorder are poorly understood.

Stress is described as a non-specific neurobiological response to environmental demands, which implies a constellation of physiological and psychological changes (McEwen, 2007; Selye, 1955). Conceptually, the stress response has evolved into a process called “Allostasis”, which is defined as the adaptive processes that maintain stability through stressful conditions (McEwen, 2000). In a stressful context, allostasis involves normal physiological responses that enable adaptation to the threat. One of the most significant of these responses is the activation of the limbic-hypothalamic-pituitary-adrenal (L-HPA) axis, which in turn leads to the secretion of stress hormones, such as the glucocorticoid cortisol (Sapolsky et al., 2000). Additionally, a normal stress response includes changes in attentional processes to handle the threat experience successfully (Rued et al., 2019; Sänger et al., 2014). In this sense, the initial phase of the stress response involves an enhanced vigilance that facilitates detecting possible threats (de Kloet et al., 2005), which may also detriments of higher-order cognitive function (van Marle et al., 2009). Several studies propose that acute activation of the L-HPA axis plays a role in shifting attentional balance from a task-directed mode, controlled by the prefrontal cortex (PFC), to a sensory-vigilance mode, regulated by the amygdala and other threat-sensitive regions (Arnsten, 2009; Bishop, 2007; Davis and Whalen, 2001).

A central modulator of the stress-induced attentional switches from ‘Top-Down’ to ‘Bottom-up’ is the locus coeruleus-noradrenergic system (LC-NE) (Nieuwenhuis et al., 2005). Under acute stress, an increase in the baseline activity of the noradrenergic system above optimal levels results in a disruption of PFC processing, generating a disruption of higher-order PFC abilities (Arnsten, 2009; Aston-Jones and Cohen, 2005; Xing et al., 2016). Furthermore, elevated LC-NE activity increases basolateral amygdala firing rates associated with stimulus-directed attention (Davis and Whalen, 2001; Giustino et al., 2020). Interestingly neuronal recordings from primate LC showed an inverted-U relationship between two different modes of LC related to attentional control (Aston-Jones et al., 1997a; Aston-Jones and Waterhouse, 2016; Rajkowski et al., 1994). Therefore, non-optimal levels of LC tonic discharge in attentional tasks are associated with poor performance (Rajkowski et al., 1994; Wainstein et al., 2021). Attentional performance is optimal under moderate LC tonic activity and prominent phasic LC activation following goal-relevant stimuli (phasic LC mode) (Aston-Jones et al., 1997b; Clayton et al., 2004). Acute stress is related to increased levels of tonic LC activity, which decreases the phasic LC evoked activity, worsening performance in attentional tasks (Aston-Jones et al., 1994; Aston-Jones and Cohen, 2005). Stimulus-driven attention in stressful situations may be related to deficient inhibitory phasic input from LC on other cortical areas involved in the orienting response to distracting stimuli (Corbetta and Shulman, 2002; Pessoa et al., 2003). However, the dynamics of the LC-NE system in humans under acute stress are unclear due to the difficulty of measuring the system’s activity in a non-invasive manner.

Importantly, pupil diameter has been directly related to activity in the LC, which sends projections to the autonomic nuclei controlling the pupils. Several studies have shown a close relationship between the type of LC activity and pupillary activity (Joshi et al., 2016; Liu et al., 2017; Murphy et al., 2014). In this regard, Rajkowski et al. (1993) observed a co-variation between the tonic activity of noradrenergic neurons in the LC and basal pupillary activity (Rajkowski et al., 1993). Subsequently, other studies have shown that spontaneous and drug-induced drowsiness and other low-activation states, characterized by low tonic LC activity, are accompanied by a decrease in basal pupil diameter (Hou et al., 2005; Morad et al., 2000). Conversely, noradrenergic drugs that increase activation and tonic LC activity also increase basal pupil diameter (Phillips et al., 2000). Finally, a large number of studies have shown that task processing is accompanied by rapid and dramatic pupillary dilation (Beatty, 1982a, Beatty, 1982b, Einhäuser et al., 2008, Richer and Beatty, 1987). These results are consistent with the emergence of a phasic LC response to task-relevant events (Aston-Jones et al., 1994). Therefore, an indirect and non-invasive technique for recording the LC-NE system and its role in modulating cortical processing and changes in attentional biases is pupillary response (Joshi et al., 2016; Murphy et al., 2014; Varazzani et al., 2015) and electroencephalography (EEG) analysis (Nieuwenhuis et al., 2005).

Psychosocial stress is a critical factor that negatively impacts our general wellbeing and daily activities. However, only a few EEG research have addressed the link between experiences of psychosocial stress and attentional processing (Jiang et al., 2017; Palacios-García et al., 2021; Qi et al., 2018; Qi and Gao, 2020; Sänger et al., 2014). ERP studies demonstrate that acute stress increases the amplitude of early components, such as N1 and Mismatch Negativity (MMN), while decreasing the amplitude of later components, such as P3 (Elling et al., 2011; Qi et al., 2018; Shackman et al., 2011). These pieces of evidence suggest that under threatening social conditions, stimulus-driven processes are enhanced, and top-down control declines (Elling et al., 2011; Sänger et al., 2014; Shackman et al., 2011). Nevertheless, the electrophysiology of attentional shifting and the activity of the LC-NE system have not been performed. In this context, the impact of acute psychological stress on the LC-NE system and its relationship with changes in attentional control is unclear.

This research aimed to analyze evoked potential (ERP) temporal dynamics to distinguish, under normal allostatic conditions, the impact of psychosocial stress on attentional control. We recorded subjective stress/anxiety, salivary cortisol, and heart rate measurements at different experiment stages to observe the psychological and physiological stress response. The relationship between the activity of the LC-NE system and changes in the attention process was obtained indirectly by a parallel recording of pupil phasic response and the ERPs generated by the auditory oddball before and after acute psychosocial stress.

We hypothesize that under acute psychosocial stress, the L-HPA axis increases the tonic activity of LC, going from an attentional control governed by the prefrontal cortex to an attentional control governed by the amygdala and other threat-sensitive regions. These changes will be reflected in an increase in the amplitude of early potentials (N1) and a decrease in the amplitude of late potentials (P3) and pupillary dilation associated with relevant stimuli for the cognitive task.

## 2 Materials and methods

### 2.1 Participants

Sixty-two male volunteers (mean age = 21,58 years [SD = 3,25]) participated in the experiment. All participants were recruited from the Pontifical Catholic University of Chile student population. All volunteers were right-handed, had normal or corrected-to-normal hearing, and had no reported neurological, psychiatric, or endocrine disease history. Additionally, no subject reported the use of psychoactive drugs or corticosteroids. High significant anxiety and stress levels were used as exclusion criteria for volunteers with allostatic overload and greater vulnerability to stress.

Additionally, participants were instructed to refrain from nicotine, caffeine, and alcohol and taking naps before the experiment. Subjects were randomly placed into the control group (CONTROL: N = 31 mean age = 22,35 years [SD = 3,58]) or stress group (STRESS: N = 31 mean age = 20,81 years [SD = 2,73]). Before the experiment, the subjects read and, when they agreed, signed an informed consent form approved by the ethical committee of the Pontifical Catholic University of Chile.

### 2.2 Stress induction protocol

**Fig. 1:**
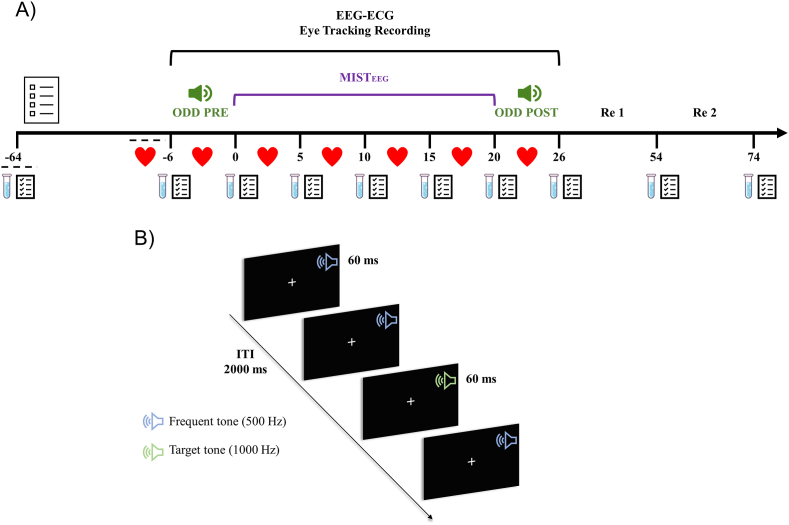
**Experimental Design.** A: Schematic of the temporal course of the experiment. The numbers represent the acquisition time of the salivary cortisol samples and the application of the stress scale (VAS) and abbreviated anxiety state inventory (short-STAI). Hearts represents 5 min of ECG recordings, and the dashed line indicates the sample used as a baseline for each measurement. Auditory oddball before (ODD PRE) and auditory oddball after (ODD POST) of acute psychosocial stress are represented by green speakers. The stress procedure involved an EEG-compatible modified Montreal imaging stress task (MISTEEG). The complete protocol lasted 20 min and was divided into (1) Anticipatory stress and (2) Arithmetic and social stress. B: Auditory oddball paradigm. The infrequent stimuli: 1000 Hz tone (target tone), and the frequent stimuli: 500 Hz tone (standard tone). Stimuli were presented semi-randomized for 60 ms with an inter-trial interval (ITI) of 2000 ms. The oddball task required pressing “space” when a target tone appeared. (For interpretation of the references to color in this figure legend, the reader is referred to the Web version of this article.)

The stress protocol consisted of an EEG-compatible modified version of the Montreal imaging stress task (MISTEEG). Previous studies showed that MIST would increase L-HPA axis activity and subjective stress/anxiety levels through a negative social evaluation (Dedovic et., al 2005; Geva et al., 2014; Lederbogen et al., 2011). During MISTEEG, subjects kept their heads in a forehead/chin rest (S.R Research Ltd.). They solved arithmetic problems presented on the computer screen, using only the index finger to answer (Dedovic et al., 2005). The complete MISTEEG stress protocol lasted 20 min and was divided into two stages: (1) Anticipatory stress (Anxiety) and (2) Arithmetic and social stress (Fig. 1A). In the anticipatory stress stage, participants were informed that their “arithmetic intelligence” was going to be studied, and only good performance (>80%) would be valid for the study. Additionally, two confederates were presented (assistant and co-investigator). The participant was notified that an assistant is supervising his performance in a second monitor while the co-investigator will be with him (the participant) throughout the arithmetic task. The investigator and co-investigator remained behind the participant during arithmetic and social stress, observing performance. The co-investigator gave a negative evaluation after an incorrect response and reiterated the requirements of the study. The phrases used to generate a negative social evaluation and threats to self-image were used in 5 periods and are detailed in S1 Table. The control condition was similar in physical and mental efforts of the stress condition without the stress-inducing component of the MISTEEG (S1 Fig).

### 2.3 Attentional task: auditory oddball

Auditory oddball is composed of two types of stimuli, infrequent and frequent. The infrequent stimuli consist of a tone sampled at 1000 Hz (target tone), whereas the frequent stimuli are a tone sampled at 500 Hz (standard tone). While the subjects looked at a white fixation cross on a black screen, auditory stimuli were presented binaurally at the 60-dB level. For each trial, auditory stimuli were presented semi-randomized for 60 ms with an inter-trial-interval (I.T.I) of 2000 ms (Fig. 1B). Each oddball block contained 200 trials; 20% had a target tone, and 80% included a frequent tone (no consecutive target tone was generated). The oddball task required pressing the “space key” when a target tone appeared and " not responding " when a standard tone was generated. Participants were instructed to respond as quickly as possible, avoiding errors. Reaction time and correct target detection were evaluated as behavioral measures recorded.

### 2.4 Experimental design

Control and stress groups were exposed to the following experimental design. Once they arrived at the laboratory, 64 min before induction of MISTEEG, participants completed a battery of psychological questionnaires to measure baseline anxiety and stress levels. After installing EEG and ECG, the first auditory oddball is applied (ODD PRE). Following the oddball paradigm, the acute psychosocial stress protocol was used, where a practice stage (4 min) preceded the control and stress of MISTEEG. In this period, arithmetic exercises were presented with five levels of difficulty. The co-investigator and computer program generated high-performance expectations and negative social evaluations only in the stress group. After the end of MISTEEG, a second auditory oddball (ODD POST) was applied to both groups. In the stress condition, subjects perform the oddball task in the presence of the co-investigator, who remains silent, out of the participant's field of vision. After completing all tasks, electrodes were removed, and participants stayed alone in the experimental room for two recovery periods (RE1: 54 min and RE2: 74 min after starting MISTEEG). A salivary cortisol sample and personal stress/anxiety levels were recorded (Fig. 1A).

### 2.5 Measurements of the physiological and psychological stress response

#### 2.5.1 Self-report measures

After arrival, basal symptoms of depression, stress and anxiety were assessed using the Beck Depression Inventory-II (BDI) (Beck et al., 1996), Beck anxiety inventory (BAI) (Beck et al., 1988) and Perceived Stress Scale (PSS) (Cohen et al., 1983). Additionally, state and trait anxiety were assessed by State-Trait Anxiety Inventory (Spielberger, 1983). Perceived stress at different experiment stages was evaluated using a visual analogue scale (VAS) (Geva et al., 2014). The VAS consisted of a 10-cm line with two anchor points at its extreme, set as “no stress” = 0 and “worst imaginable stress” = 10. VAS was applied at specific times relative to the beginning of MITSEEG (0 min): −64 min, −6 min, 0 min, 20 min, 26 min, 54 min and 74 min. To analyze changes in perceived stress levels throughout the experiment, stress levels obtained in −64 min (baseline) were compared with the six other samples (see Statistical Analyses section). Anxiety levels during the experiment were evaluated using a short version of the state-trait anxiety questionnaire (STAIshort) (Spielberger, 1983). Only the section corresponding to the state was used. This questionnaire contains ten items, and subjects are asked to rate the degree to which they experienced each symptom of anxiety at that moment on a 4-point Likert-type scale (1 = not at all, to 4 = very much so). The questionnaire was applied at specific times relative to the beginning of MITSEEG (0 min): −64 min, −6 min, 0 min, 20 min, 26 min, 54 min and 74 min. To analyze changes in anxiety throughout the experiment, anxiety levels obtained in −64 min (baseline) were compared with the six other samples (see Statistical Analyses section).

#### 2.5.2 Heart rate analysis

ECG activity was monitored throughout the experiment, using two external electrodes (BioSemi ActiveTwo®) positioned below the left clavicle and over the left hip. We obtained seven samples of 5 min: resting state (baseline), during the first oddball (ODD PRE), four samples during the 20-min of stress protocol (MISTEEG) and the second oddball (ODD POST). The heart rate data was obtained and calculated using Kubios software (Tarvainen et al., 2014). To analyze the physiological manipulation of the MISTEEG, the heart rate data of each experimental stage was compared with baseline heart rate (see Statistical Analyses section).

#### 2.5.3 Salivary cortisol analysis

Multiple saliva samples (Sarstedt, Germany) were collected across the experiment to quantify the impact of acute psychosocial stress protocol on cortisol. The samples were taken at specific times relative to the beginning of MITSEEG (0 min): −64 min, −6 min, 0 min, 5 min, 10 min, 15 min, 20 min, 26 min, 54 min and 74 min. Saliva samples were stored at −20 °C until the end of the study. The salivary cortisol level was determined via Cortisol ELISA (catalogue item 11-CORHU-E01-SLV; ALPCO, Salem, NH) using a standard curve of known cortisol concentration in a protein-based buffer as supplied with the assay kit, following the manufacturer's instruction. The sensitivity of the assay was 1.0 ng/ml. Optical density values were measured at 450 nm using a microplate reader (Tecan GENios™, Tecan Group Ltd., Austria). To analyze the physiological manipulation of the MITSEEG, cortisol levels obtained in −64 min (baseline) was compared with the nine other samples. The values were expressed in ng/mL (see Statistical Analyses section).

### 2.6 Pupil recording and analysis

#### 2.6.1 Pupil recording

Pupil diameter data were acquired with Eyelink 1000 (SR Research Ltd., Mississauga, Ontario, Canada), sampled at a 500 Hz sampling rate. The system was calibrated using the Eyelink 9-point automated calibration routine before each auditory oddball. Participants were placed at a viewing distance of 60 cm from the computer screen using a forehead/chin rest (SR Research Ltd.). The computer screen had 27 inches with a 1920 × 1080 resolution. Luminescence was measured using a lux-meter positioned in the same chin-rest used by the subjects at the same distance from the screen. Illuminance changes were on the order of >1 lux (as a reference, a change from white to black screens, in our setup, is associated with a drop of ∼100 lux).

#### 2.6.2 Pupil analysis

For analysis, pupil diameter data of the left eye was chosen. First, periods of blinks were detected by the Eyelink software. Pupil data surrounding blinks were removed from the time series used in the analyses. Pupil diameter during these periods was estimated using cubic spline interpolation, implemented through Matlab function spline. The signal was smoothed by a bandpass Butterworth filter between 0.025 Hz and 4 Hz. The outliers, defined as periods of pupil change (derivative function) higher than three standard errors from the mean, were discarded. Finally, the analysis did not consider all trials with more than 50% of missing data (due to blinks or outliers). After filtering and outlier removal, the pupil time-series were normalized by a z-score separately for each trial.

After filtering and outlier removal, the pupil time-series were normalized by a z-score, separately for each trial.

To quantify the pupillary phasic response in each oddball, the pupil diameter data were epoched from −2000 to 4000 ms relative to the target stimulus onset (time interval from −2000 to 0 ms was used as a baseline). Afterwards, pupil dilation from baseline was independently calculated for each trial (Wainstein et al., 2017).

### 2.7 EEG recording and analysis

#### 2.7.1 EEG recording

The EEG data were recorded using a Biosemi ® ActiveTwo system (Biosemi, Amsterdam, Netherlands), with a 2048 Hz sampling rate from 64 Ag/AgCl electrodes mounted according to the extended 10/20 extended system. During the recording, impedances were kept below 20 kΩ. Eye movements were controlled with two additional derivations (vertical electrooculogram, VEOG, and horizontal electrooculogram, HEOG). For ERP analyses, only correct responses to target and standard stimuli were considered.

#### 2.7.2 Signal pre-processing for ERP and Factorial Mass Univariate Test (FMUT) analysis

The data were pre-processed and analyzed with MATLAB (MathWorks, Inc., Natick, MA) using EEGLAB toolbox (version 2019.0) (Delorme and Makeig, 2004) and ERPLAB (version 7.0.0) (Lopez-Calderon and Luck, 2014). For each subject, the two blocks of the oddball paradigm were concatenated. Then, the data were resampled to 1024 Hz, bandpass filtered using a Butterworth filter (half amplitude cutoffs at 0.1 and 100 Hz, 12 dB/octave roll-off), and the re-referenced average of the left and right mastoids.

Continuous data were epoched into 2000 ms segments (200 ms before and 1800 ms after the auditory stimulus onset). Segments of EEG were visually inspected to identify and remove bad channels and muscular activity artefacts. Blinks and eye movement artefacts were placed and manually removed using independent component analysis (ICA) (Delorme and Makeig, 2004; Jung et al., 2000a; Jung et al., 2000b). After ICA, removed channels were interpolated using spherical-spline interpolation, as implemented in EEGLAB. The data was filtered using a Butterworth filter (low-pass filter 40 Hz, 12 dB/octave roll-off). The remaining artefacts were automatically rejected using the moving peak-to-peak algorithm (Lopez-Calderon and Luck, 2014) (with a voltage threshold of ±100 mV, moving windows full width of 200 ms and window step of 50 ms) in epochs from −200 to 800 ms relative to the auditory stimulus onset.

For ERPs elicited by the target tone in auditory oddball before stress (ODD PRE) an average of 1.7% of epochs were rejected from the control group, leaving an average of 38.16 (SD = 4.4) epochs per participant. An average of 1.4% of epochs were rejected from the stress group, leaving an average of 39.45 (SD = 1.9) epochs per participant. For ERPs elicited by the target tone in auditory oddball after stress (ODD POST) an average of 0.7% of epochs were rejected from the control group, leaving an average of 38.81 (SD = 3.6) epochs per participant. An average of 1.0% of epochs were rejected from the stress group, leaving an average of 39.61 (SD = 0.95) epochs per participant. For ERPs elicited by the frequent tone in auditory oddball before stress an average of 1.5% of epochs were rejected from the control group, leaving an average of 152.80 (SD = 17.8) epochs per participant. An average of 1.1% of epochs were rejected from the stress group, leaving an average of 158.5 (SD = 5.5) epochs per participant. For ERPs elicited by the frequent tone in auditory oddball after stress an average of 1.6% of epochs were rejected from the control group, leaving an average of 153.40 (SD = 16.4) epochs per participant. An average of 0.5% of epochs were rejected from the stress group, leaving an average of 159.10 (SD = 2.93) epochs per participant. The pre-stimulus window of 200 ms was used to correct baseline activity in each trial.

#### 2.7.3 Factorial Mass Univariate Test (FMUT) analysis

For exploratory ERP analysis, the factorial univariate test was performed using the FMUT toolbox (Groppe et al., 2011). The factorial analysis permitted comparison of each electrode of interest and time point of interest, allowing for more temporally and topologically focal exploration of ERPs per a priori specifications. Permutation-based mass univariate cluster corrections were applied to analyses (Groppe et al., 2011) to account for multiple comparisons with the following parameters: Factors time and group (2 × 2),10.000 permutations for specific time window [0–703 ms], using an alpha of 0.05, and average neighbours channels of 4 (electrodes considered neighbours for clustering). Factorial Mass Univariate ERP Toolbox [Computer software]. Available from: https://github.com/ericcfields/FMUT/releases).

#### 2.7.4 ERP analysis

A statistical analysis based on regions of interest (ROIs) was performed to identify the stress effects in areas related to attentional processing. Each ROI was the average of five electrodes aligned with the z-axis: frontal (AFZ, Fz, FCZ, F1 and F2) and posterior (CPZ, Pz, POZ, P1 and P2) electrodes. The maximum amplitude point of two temporary windows (100–200 ms and 300–500 ms) related to N1 and P3s was used to define temporary analysis windows. The analysis window (±50 ms and) was determined for each component from the amplitude peak. Then, the ERP stress effect was calculated as the difference between ERPs elicited by the target stimulus in the auditory oddball after stress (ODD POST) and the auditory oddball before stress (ODD PRE). The Mismatch Negativity (MMN) was generated by subtracting the signal evoked by the standard stimulus from the signal evoked by the target stimulus. A temporal analysis window of 120–300 ms was used, focusing on a frontal ROI (AFZ, Fz, FCZ, F1, and F2) (Kärgel et al., 2014; Näätänen et al., 2007). The MMN components generated before and after the application of the MISTEEG were compared to assess the effect of stress on MMN.

### 2.8 Statistical analyses

#### 2.8.1 Physiological and psychological analysis

To analyze the physiological and psychological changes generated by MISTEEG over time, we employed two-way ANOVA with repeated measures, incorporating time and group as factors, using an alpha value of P < 0.05 as the level of statistical significance. We used Bonferroni multiple comparison test to compare each measure within the same group to a baseline state.

#### 2.8.2 Behavioral analysis

We analyzed performance using a two-way ANOVA with repeated measures, incorporating time and group as factors, and considering an P < 0.05 as statistically significant. Additionally, we analyzed the difference score (ODD POST minus ODD PRE) using the non-parametric Mann-Whitney *U* test. All p-values reported were two-tailed and considered significant if they were less than 0.05.

#### 2.8.3 Pupil analysis

To assess reliable differences in pupil size responses associated with the target stimulus between ODD PRE and ODD POST, we employed the Wilcoxon signed-rank test using the Matlab function “signrank”. We set the significant difference level to P < 0.05.

#### 2.8.4 ERP analysis

To compare the average amplitude of each ROI before and after MISTEEG, we analyzed data within pre-determined analysis windows of N1 (152 ± 50 ms), MMN (220 ± 100 ms), and P3s (375 ± 50 ms). We used ANOVA with repeated measures, incorporating time and group as factors, with a significance level of P < 0.05. Post-hoc tests were performed using Bonferroni multiple comparisons.

#### 2.8.5 Permutation test and cluster correction

The mass univariate approach for analyzing ERPs involves conducting ANOVA for each time point and electrode of interest, followed by a correction method to control the Type I error. Our study utilized a permutation-based cluster mass correction to control Type I error. This correction method tests clusters for significance rather than individual electrodes or time points. Initially, a threshold for cluster inclusion is defined as the F-value that would be significant without correction. Spatially and temporally adjacent points that exceed this threshold are considered a cluster. All F-values in a cluster are summed to calculate the cluster mass statistic. The null distribution for the cluster mass statistic is estimated through a permutation approach, and observed clusters that exceed the 1 - α percentile of the distribution are considered significant. To calculate effects in factorial designs, FMUT reduces the data to the most straightforward design that can calculate an equivalent test (Welch, 1990). FMUT reduces data via the following steps: Average (within subjects and levels of the other factors) across all within-subjects factors not involved in an effect. For interaction effects, iteratively subtract (within subjects and levels of the other factors) across any factors with two levels. Then a one-way permutation ANOVA is conducted.

#### 2.8.6 Correlational analysis

To analyze the correlation between the effects of MISTEEG and physiological and electrophysiological variables in all subjects, we measured the differences in pupillary dilation and P3b amplitude before and after the MISTEEG, as well as heart rate response and salivary cortisol levels. For heart rate response, we calculated the difference between the 20-min of the MISTEEG average and the average of the 5 min of resting state (baseline). We used the difference between the cortisol level 25 min after the MISTEEG and the baseline salivary cortisol level. Finally, we performed a Spearman correlation test with a significance level of P < 0.05.

Statistical analyses were performed using the GraphPad Prism program (version 7, GraphPad Software, San Diego, CA). Spearman's correlation with the R script calculated the relationship between variables.

## 3 Results

### 3.1 Participant

Pupillary data from fifteen participants were excluded from further analysis for technical reasons (i.e., poor signal quality pupillary and unexpected recording failures). A final sample of forty-seven participants for pupillary analyses (CONTROL: N = 18 mean age = 21,74 years [SD = 3160], STRESS: N = 29 mean age = 20,54 years [SD = 2186]). The excluded individuals did not differ significantly from the final cohort in any of the baseline characteristics.

The evaluation of depressive symptoms (BDI) and anxiety levels (BAI, STAI STATE) did not show significant differences between the control and stress groups. Additionally, no significant difference in anxiety traits (STAI TRAIT) and stress perceived (PSS) were found (Table 1).

**Table 1:** Summary of the characteristics and levels of anxiety/stress of the control and stress groups.

	CONTROL		STRESS		
	MEAN	SD	MEAN	SD	P-value
PSS	23,16	8,35	20,74	10,10	0.308
STATE STAI	13,16	6,73	11,81	9236	0.511
TRAIT STAI	21,10	9,48	20,00	10,13	0.661
BDI	7226	5,04	6645	5037	0.651
BAI	11,23	8,26	13,23	9524	0.380
AGE	22,35	3,58	20,81	2738	0.060

Table 1. PSS: Perceived Stress Scale, STAI: State-Trait Anxiety Inventory, BDI: Beck Depression Inventory-II, BAI: Beck anxiety inventory. SD=Standard deviation.

### 3.2 Physiological stress response

We compared heart rate and salivary cortisol levels in different experimental stages to evaluate the physiological response to stress induction.

**Fig. 2:**
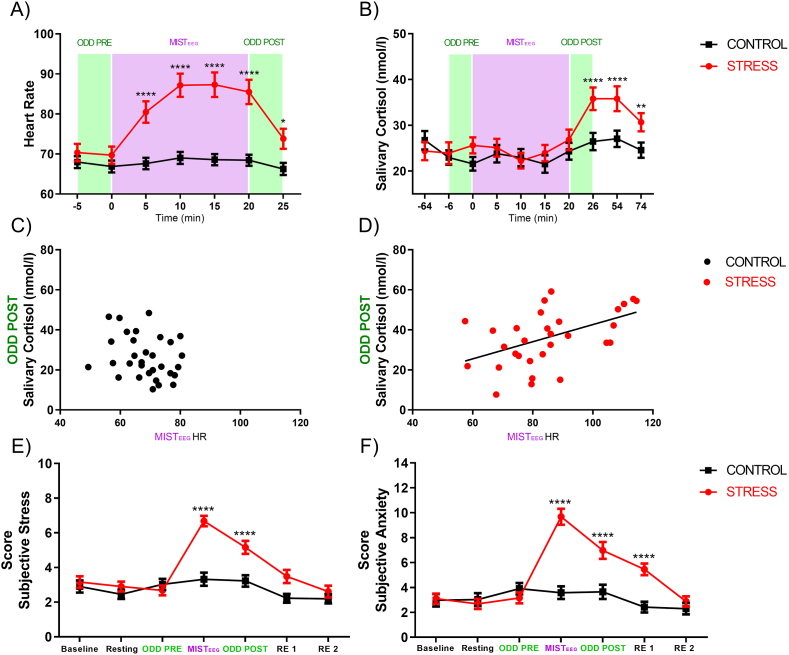
**Physiological and psychological response to acute psychosocial stress.** Simultaneous measures for heart rate (A) and salivary cortisol (B). Time 0 represents the start of MISTEEG. The Green column represents auditory oddballs, and the purple columns represent MISTEEG. Relationship between heart rate (20 min average of MISTEEG) and salivary cortisol (ODD POST) for the control group (C) and stress group (D). Simultaneous measures for subjective stress (E) and anxiety perceived (F). RE 1: the first recovery period (54 min after the start of MISTEEG). RE 2: the second recovery period (74 min after the start of MISTEEG). The first sample was a baseline for each physiological and psychological measure. Error bars ± S.E.M. and asterisks indicate statistically significant differences (*: p < 0.05). (For interpretation of the references to color in this figure legend, the reader is referred to the Web version of this article.)

First, to identify if there were significant differences between groups, we compared the baseline heart rate and baseline salivary cortisol levels. We found no differences neither for heart rate (Fig. 2A, mean ± S.D., Control: 67.92 ± 8.21, Stress: 70.35 ± 12.03 P > 0.05), nor for basal cortisol (Fig. 2B, mean ± S.D., Control: 26.73 ± 11.31, Stress: 24.53 ± 11.53 P > 0.05).

However, when comparing heart rate levels at different stages of the experiment ANOVA with repeated measures showed a significant main effect for group [F (1, 60) = 16.07 P < 0.001, η^2^p = 0.21] and a significant main effect for time [F (6, 360) = 53.75 P < 0.0001, η^2^p = 0.47]. Even more, the interaction between time and group [F (6, 360) = 36.57 P < 0.001, η^2^p = 0.37] was detected. Bonferroni's multiple comparisons tests showed that MISTEEG significantly increases heart rate only in the stress group in relation to the resting state. This effect lasts until the end of the second oddball (mean ± S.D., Resting: 70.35 ± 12.03; MISTEEG 5 (min): 80.49 ± 14.89 P < 0.0001, MISTEEG 10 (min): 87.17 ± 16.09 P < 0.0001; MISTEEG 15 (min): 87.30 ± 17.06 P < 0.0001, MISTEEG 20 (min): 85,51 ± 16.91 P < 0.0001, ODD POST: 73.80 ± 13.97 P < 0.05) (Fig. 2A).

Similar results were observed in salivary cortisol levels. ANOVA with repeated measures analysis showed significant main effects for time [F (9, 540) = 13.83 P < 0.0001, η^2^p = 0.18]. Additionally, the interaction between time and group [F (9, 540) = 5.005 P < 0.0001, η^2^p = 0.07] was detected. Bonferroni's multiple comparisons test showed that 26 min after the start of MISTEEG, only the stress group significantly increased the level of salivary cortisol in relation to the baseline level (mean ± S.D., Baseline: 24.46 ± 11.54; ODD POST: 36.25 ± 13.98 P < 0.0001, Recovery 1 (54 min): 36.31 ± 15.31 P < 0.0001; Recovery 2 (74 min): 31.093 ± 11.16 P < 0.01) (Fig. 2B).

**Fig. 3:**
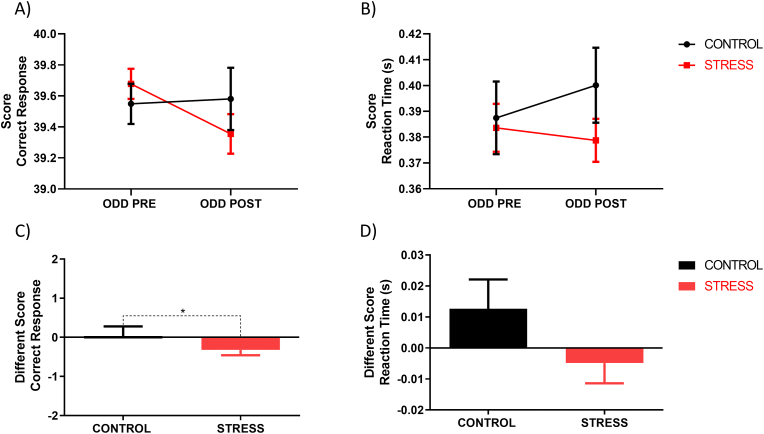
**Effect of acute psychosocial stress on performance.** Correct responses (A) and latency of correct responses (B) for ODD PRE and ODD POST. The difference score of correct responses (C) and latency of correct responses (D) for the control and stress groups. Error bars ± S.E.M. and asterisks indicate statistically significant differences (*: p < 0.05).

Subsequently, we asked ourselves if there was a relationship between the cardiac response observed during MISTEEG and the salivary cortisol levels observed in the ODD POST stage. Pearson correlation showed a positive correlation between variables only in the stress group (r = 0.49, N = 31, P < 0.01) (Fig. 3D).

### 3.3 Psychological stress response

To evaluate the psychological effects of stress induction, we analyzed the state of anxiety and subjective stress at different experiment stages.

The analysis of baseline stress perceived showed no differences between groups (Fig. 2E, mean ± S.D., Control: 2.90 ± 1.94, Stress: 3.16 ± 1.88 P > 0.05). Additionally, no significant differences in the baseline state of anxiety were found (Fig. 2F, mean ± S.D., Control: 2.97 ± 2.77, Stress: 3.10 ± 2.26 P > 0.05).

However, when comparing stress-perceived levels at different stages of the experiment ANOVA with repeated measures showed a significant main effect for group [F (1, 60) = 10.82 P < 0.01, η^2^p = 0.15] and a significant main effect for time [F (6, 360) = 28.95 P < 0.0001, η^2^p = 0.32]. Additionally, the interaction between time and group [F (6, 360) = 12.86 P < 0.0001, η^2^p = 0.17] is observed. Bonferroni's multiple comparisons tests showed that only the stress group significantly increases the level of subjective stress regarding baseline level (mean ± S.D., Baseline: 3.16 ± 1.88; MISTEEG: 6.67 ± 1.68 P < 0.0001; ODD POST: 5.16 ± 2.08 P < 0.0001) (Fig. 2E).

Similar results were observed in the state of anxiety levels. ANOVA with repeated measures showed a significant main effect for group [F (1, 60) = 10.31 P < 0.01, η^2^p = 0.14] and a significant main effect for time [F (6, 360) = 35.82 P < 0.0001, η^2^p = 0.37]. Even more, the interaction between time and group [F (6, 360) = 25.73 P < 0.0001, η^2^p = 0.30] was detected. Bonferroni's multiple comparisons tests showed that only the stress group significantly increases the level of anxiety in relation to the baseline level (mean ± S.D., Baseline: 3.09 ± 2.25; MISTEEG: 9.67 ± 3.59 P < 0.0001; ODD POST: 6.96 ± 3.77 P < 0.0001; RE 1: 5.45 ± 2.60 P < 0.0001) (Fig. 2F).

Subsequently, we observe the relationship between the state of anxiety and the stress perceived during the second auditory oddball (ODD POST). Spearman correlation showed a positive association between variables in both groups. However, the relationship in the stress group was stronger (Control: r = 0.42, N = 31, P < 0.05, Stress: r = 0.59, N = 31, P < 0.001) (S4 A,B Fig).

### 3.4 Behavioural results

Performance analysis using ANOVA with repeated measures did not detect significant differences in correct response and reaction time (Fig. 3A and B). However, when comparing the difference scores between groups (ODD POST minus ODD PRE), a significant decrease in the performance of the stressed subjects was observed (mean ± Control: 0.03 ± 1.37; Stress: −0.32 ± 0.74; Mann-Whitney *U* test, P < 0.05) (Fig. 3C). In contrast, no significant differences were found in reaction times (mean ± Control: 0.012 ± 0.052; Stress: −0.004 ± 0.036; Mann-Whitney *U* test, P > 0.05) (Fig. 3D).

### 3.5 Pupillary results

**Fig. 4:**
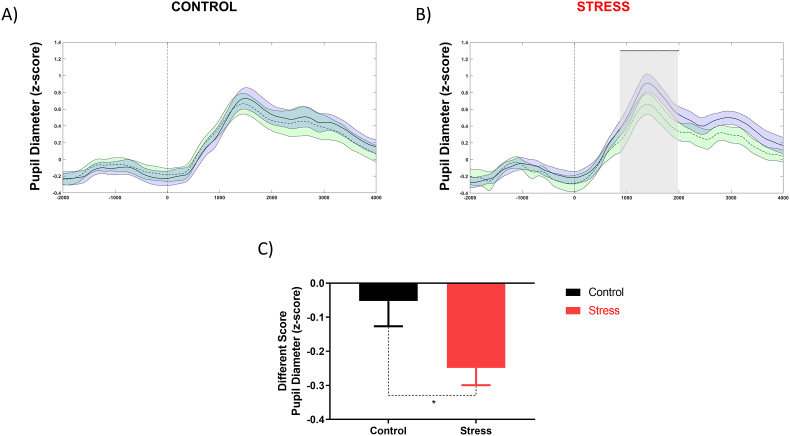
**Effect of acute psychosocial stress in pupillary phasic response.** Pupil diameter elicited by target tone for control and stress group. In blue ODD PRE and green ODD POST condition. The shaded area defines the temporal windows in which an independent permutation test detected significant positive differences between the stress group's ODD PRE and ODD POST conditions (p < 0.01). Data expressed in z-scores. (For interpretation of the references to color in this figure legend, the reader is referred to the Web version of this article.)

The Wilcoxon signed-rank test showed a significant reduction in pupil dilation only in the stress group. The reduction of the pupillary response associated with the target stimulus was observed in a time window between 882 and 1988 ms (P < 0.01) (Fig. 4 A, B). Analysis of different scores between groups (ODD POST minus ODD PRE) showed that the reduction in pupillary diameter was significantly greater in the stress group. (mean ± Control: −0.05 ± 0.31; Stress: −0.24 ± 0.27; Mann-Whitney *U* test, P < 0.05) (Fig. 4C).

### 3.6 Electrophysiological results

#### 3.6.1 Cluster-based permutation analysis

**Fig. 5:**
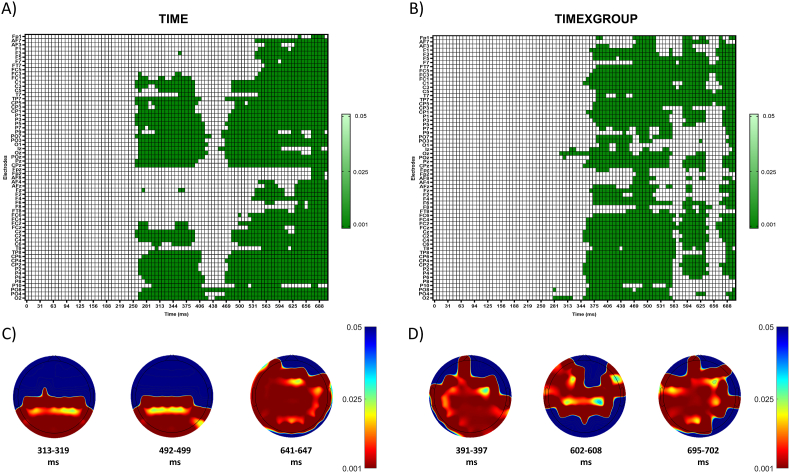
**Cluster topography relative to factorial univariate test.** Raster diagram illustrating significant main effect for the factor time (A) and timexgroup interaction (B) according to permutation test based on the cluster mass statistic. Each green-colored lectrode/timepoint represents a significant p-value (p < 0.05). White rectangles indicate electrodes/time points at which no significant effect was found. Note that the electrodes are organized along the y-axis somewhat topographically. Electrodes on the left and right sides of the head are grouped on the figure's top and bottom, respectively. Midline electrodes are shown in the middle. Within those three groupings, the y-axis top-to-bottom corresponds to the scalp anterior-to-posterior. Representation of changes in the cluster topography at different latencies for factor time (C) and timexgroup interaction (D). The heat map represents the distribution of p-values for the 64 electrodes. (For interpretation of the references to color in this figure legend, the reader is referred to the Web version of this article.)

The mass univariate permutation approach to the current factorial analyses showed a significant main effect for the factor time in the same temporal window reported for the P3s associated with the target stimulus, where parietal and occipital sites play a prominent role (P = 0.001). Latencies for the clusters range from 258 to 703 ms. They comprehend electrodes from all areas (Fig. 5A, C and S2 Table). Besides, there was a significant interaction effect between group and time in the same temporal window reported for the P3s (P = 0.002). Specifically, the cluster ranges from 281 to 703 ms. (Fig. 5B, D and S2 Table). Additionally, a greater temporal mass of the cluster was found between 305 and 359 ms, 570–586 ms, 672–703 ms (S2 Fig A), and between 383 and 508 ms, 594–602 ms, 695–703 ms (S2 Fig B).

**Fig. 6:**
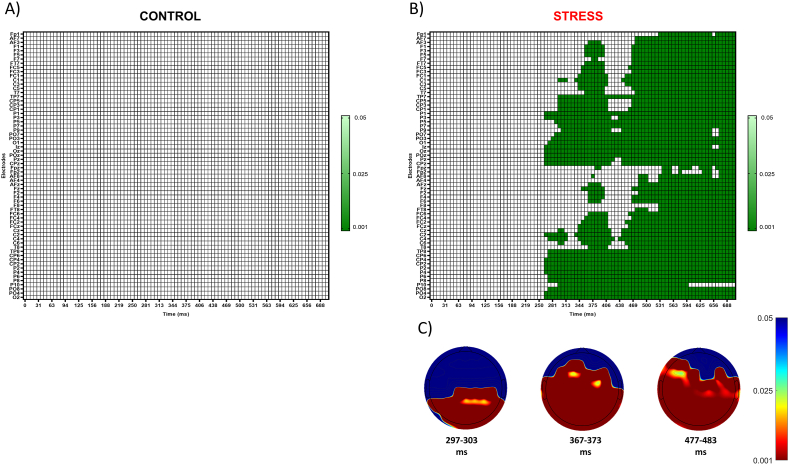
**Cluster topography relative to group-by-time pair-wise comparisons.** Raster diagram of ERP amplitude differences between ODD PRE and ODD POST for control (A) and stress group (B) according to permutation test based on the cluster mass statistic. Each green-colored lectrode/timepoint represents a significant p-value (p < 0.05). White rectangles indicate electrodes/time points at which no significant effect was found. Note that the electrodes are organized along the y-axis somewhat topographically. Electrodes on the left and right sides of the head are grouped on the figure's top and bottom, respectively. Midline electrodes are shown in the middle. Within those three groupings, the y-axis top-to-bottom corresponds to the scalp anterior-to-posterior. Representation of changes in the cluster topography at different latencies (C). The Heat map represents the distribution of p values for the 64 electrodes. (For interpretation of the references to color in this figure legend, the reader is referred to the Web version of this article.)

Following up main effects in factorial designs, we perform pair-wise comparisons between ODD PRE and ODD POST in both groups. Cluster analysis showed a significant difference in ERP amplitude associated with the target stimulus only in the stress group (P < 0.001). Specifically, the cluster ranges from 266 to 703 ms, and spatial extent across several areas see (Fig. 6B, C and S3 Table). Additionally, a greater temporal mass of the cluster was found between 383 and 398 ms, 492–641 ms, and 672–703 ms (S3 Fig B).

#### 3.6.2 ROI analysis of the early and late ERP effects

**Fig. 7:**
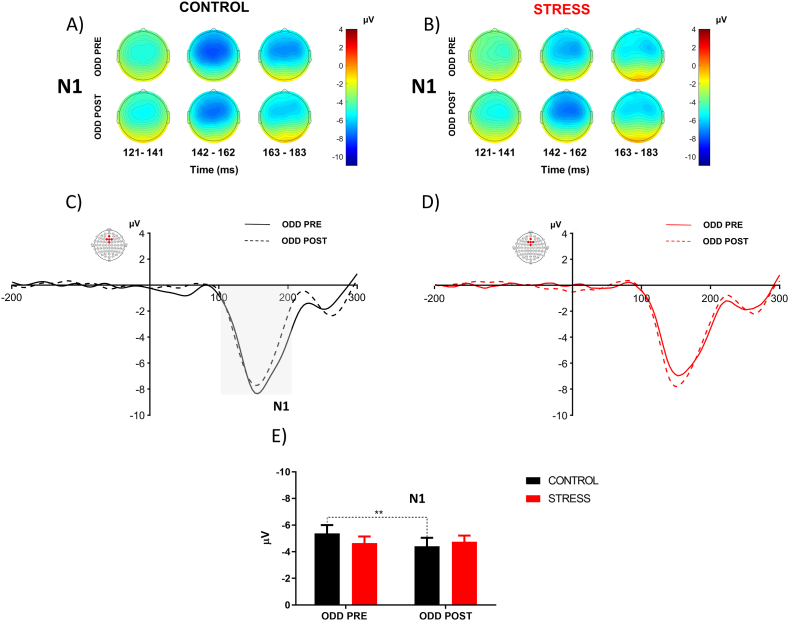
**Effect of acute psychosocial stress on N1.** Scalp distributions of N1 component associated with target tone for ODD PRE and ODD POST (A, B). N1 component elicited to target tone, the continuous line represented ODD PRE and the dashed line represented ODD POST (C, D). Average quantification of N1 amplitude for control and stress group (E, F). Error bars ± S.E.M. and asterisks indicate statistically significant differences (*: p < 0.05).

**Fig. 8:**
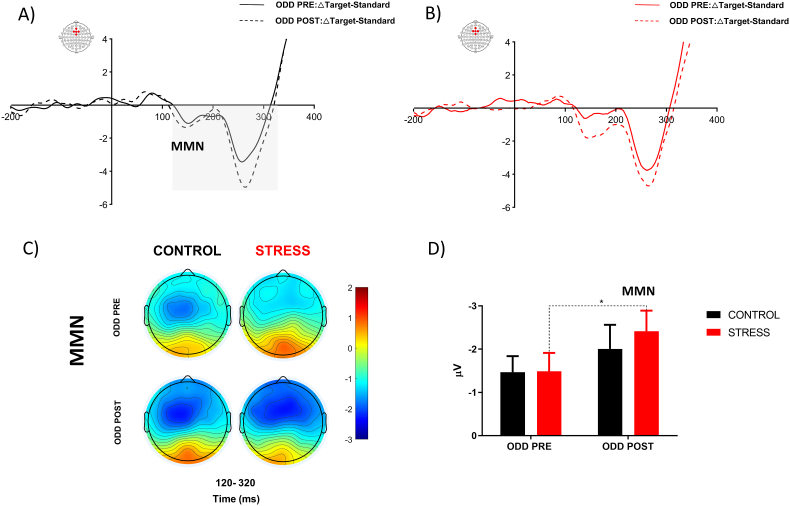
**Effect of acute psychosocial stress on MMN.** MMN obtained by subtracting the signal elicited by the target and standard stimulus, the continuous line represented ODD PRE and dashed line represented ODD POST for Control (A) and stress group (B). The scalp distributions of MMN component for ODD PRE and ODD POST (C). Average quantification of MMN amplitude for control and stress group(D). Error bars ± S.E.M. and asterisks indicate statistically significant differences (*: p < 0.05).

**Fig. 9:**
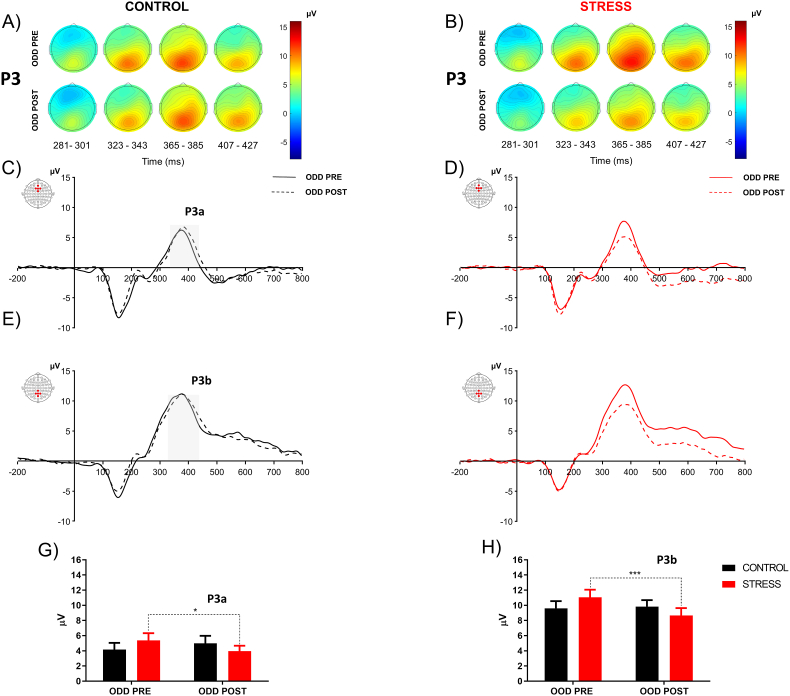
**Effect of acute psychosocial stress on P3.** The scalp distributions of P3 component associated with target tone for ODD PRE and ODD POST (A, B). P3a component (C, D) and P3b component (E, F) elicited to target. The continuous line represented ODD PRE, and the dashed line represented ODD POST average P3a and P3b amplitude quantification for the control and stress group (G, H). Error bars ± S.E.M. and asterisks indicate statistically significant differences (*: p < 0.05).

The topography of N1 (Fig. 7A and B), MMN (Fig. 8C) and 3Ps (Fig. 9A and B) are plotted for the control and stress groups. Visual analysis shows that the control group decrease the N1 component in ODD POST. Instead, the stress group showed an increase in N1 and MMN after psychosocial stress. However, stressed subjects decreased P3a and P3b amplitude in ODD POST.

To assess the impact of psychosocial stress on bottom-up processes, we evaluated temporal windows associated with N1 related to the target stimulus in frontal ROI (AFZ, Fz, FCZ, F1 and F2). Two-way ANOVA with repeated measures showed an interaction between time and group [F (1, 60) = 4.04 P < 0.05, η^2^p = 0.09]. Bonferroni's multiple comparisons test shows that the control group significantly decrease the amplitude of N1 in the second oddball (mean ± S.D., ODD PRE: −5,37 ± 3,48; ODD POST: −4,40 ± 3,58 P < 0,05) (Fig. 7C,E). Regarding the N1 amplitude associated with the standard stimulus, we observed a notable general effect over time [F (1, 60) = 38.76, P < 0.001, η^2^p = 0.39]. Bonferroni's multiple comparison tests indicated a significant reduction in N1 amplitude in both groups during the second oddball (Control: mean ± S.D., ODD PRE: −4.56 ± 2.45; ODD POST: −3.47 ± 2.05, P < 0.001 and Stress: mean ± S.D., ODD PRE: −4.12 ± 1.90; ODD POST: −3.16 ± 1.78, P < 0.001).

Additionally, to evaluate the effect of stress on involuntary attention processes, we assessed the temporal windows associated with MMN in the frontal ROI (AFZ, Fz, FCZ, F1, and F2). A two-way ANOVA with repeated measures revealed a significant main effect for time [F (1, 60) = 7.55, P < 0.001, η^2^p = 0.11]. Bonferroni's multiple comparisons tests showed that the stress group significantly increased the amplitude of MMN in the second oddball (mean ± S.D., ODD PRE: −1.48 ± 2.38; ODD POST: −2.41 ± 2.64, P < 0.05) (Fig. 8D).

Subsequently, we evaluate the effect of psychosocial stress in top-down processes using temporal windows associated with P3s components. In Frontal ROI (AFZ, Fz, FCZ, F1 and F2), two-way ANOVA with repeated measures shows the interaction between time and group [F (1, 60) = 7.284 P < 0.01, η^2^p = 0.10]. Bonferroni's multiple comparisons test show that the stress group significantly decrease the amplitude of P3a in the second oddball (mean ± S.D., ODD PRE: 5.38 ± 5.28; ODD POST: 3.95 ± 4.01 P < 0.05) (Fig. 9D,G). In the posterior ROI, two-way ANOVA with repeated measures show a time effect [F (1, 60) = 9.039 P < 0.01, η^2^p = 0.10] and interaction between time and group [F (1, 60) = 10.81 P < 0.01, η^2^p = 0.14]. Bonferroni's multiple comparisons test show that the stress group significantly decrease the amplitude of P3b in the second oddball (mean ± S.D., ODD PRE: 11,05 ± 5,27; ODD POST: 9,83 ± 5,40 P < 0,001) (Fig. 9F,H).

### 3.7 Correlational analysis of stress response

**Fig. 10:**
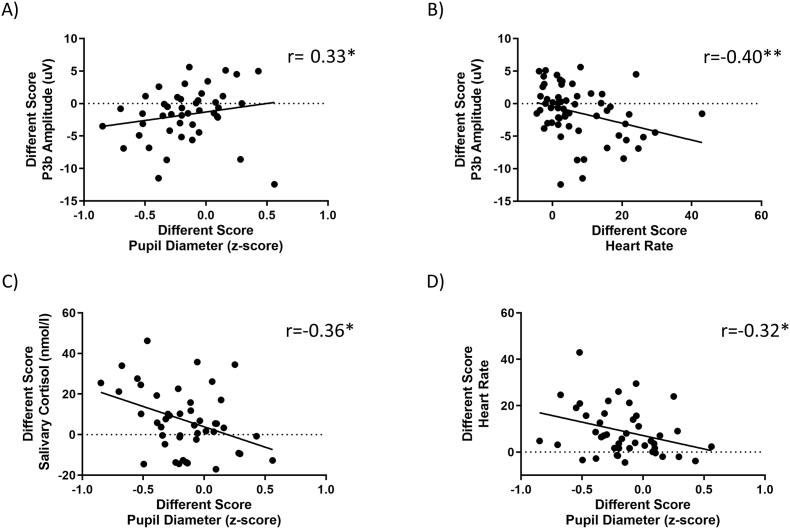
**Relationship between pupillary response, heart rate, salivary cortisol, and P3b.** Pearson's correlation between changes in pupillary response and P3b (A), P3b and heart rate (B), salivary cortisol and pupillary answer (C), heart rate and pupillary response (D). The different heart rate and cortisol salivary scores were calculated as the delta between MISTEEG and baseline levels.

Lastly, we performed multiple correlational analyses between physiological and electrophysiological variables influenced by the LC-NE system, aiming to gain a deeper understanding of the stress response and its associated electrophysiological changes during shifts in attentional control. Upon examining the interrelationships among various physiological measures, we identified several significant correlations. Specifically, a weak positive correlation (r = 0.33, p < 0.05) emerged between changes in P3b amplitude and changes in pupillary responses (Fig. 10A). Conversely, a moderate negative correlation (r = −0.40, p < 0.01) was found between changes in P3b amplitude and cardiac responses (Fig. 10B). Additionally, changes in cortisol levels and changes in pupillary responses revealed a negative correlation (r = −0.36, p < 0.01) (Fig. 10C). Lastly, a weak negative correlation (r = −0.32, p < 0.05) was observed between changes in pupillary responses and cardiac responses (Fig. 10D).

## 4 Discussion

This research aims to investigate the impact of acute psychosocial stress on attentional control and brain signatures. This study is based on a multidomain approach using self-reported, behavioural, physiological and neurophysiological measures to infer allostatic processes in contexts of negative social evaluation. We found evidence suggesting that LC phasic activity and ERP P3b decrease after stress exposure while cortisol increases. Additionally, those changes were dependent on the increases in cortisol levels. Together, these results provide evidence of the manner in which stress response modulates attentional processing, physiological responses and neuromodulatory activity.

### 4.1 Stress protocol validation

Stress protocol triggered the sympathetic and humoral response to stress. Participants of the stress group showed a significant increase in heart rate during 20 min of MISTEEG. This increase was approximately 21.4% from the baseline (Fig. 2A). Heart rate increment was accompanied by high anxiety and subjective stress (Fig. 2E and F). Further, we found that MISTEEG significantly increases salivary cortisol secretion only in the stress group, suggesting an increase in the activity of the L-HPA axis only in subjects exposed to arithmetic exercises and negative social evaluation. These results align with previous studies' findings in which psychological stress was induced by socially evaluated mental arithmetic (Dedovic et al., 2005; Pakarinen et al., 2019; Qi et al., 2017).

MISTEEG generated asynchronous change between salivary cortisol and cardiac response over time. In this line, previous psychosocial stress studies found an asynchronous response over time between salivary cortisol and heart rate (Dedovic et al., 2005; Jayasinghe et al., 2014). This evidence may reflect the different sympathetic mechanisms that modulate heart rate and cortisol release during the normal stress response (Gordan et al., 2015; Meaney et al., 1993). Moreover, the increase in salivary cortisol associated with the MISTEEG cannot be explained by the effect of the circadian cycle because the control group does not show significant changes over time. Additionally, our results show differences in physiological reactivity to psychosocial stress (Fig. 2D), which may be associated with sensitivity or latent individual vulnerability to this kind of stress.

Our results show that MISTEEG is an adequate protocol for studying psychosocial stress response.

### 4.2 Behavioural performance

Our results reveal that only the stress group decrease in accuracy after the MISTEEG (Fig. 3A,C). Since the control group was exposed to the same arithmetic problems, it is unlikely that cognitive fatigue generated by MISTEEG caused the difference in precision. In this context, our data suggest that subjects exposed to acute psychosocial stress directed attentional resources away from the cognitive task. Several studies show that oddball task performance depends on top-down attentional processes that facilitate target detection and maintain attention toward the cognitive task (Katsuki and Constantinidis, 2014; Kim, 2014). However, in threatening environments, a normal stress response involves increased alertness that enables the detection of potential threats and prompts quick fight or flight reactions (de Kloet et al., 2005). This attentional state is associated with elevated activity of the LC-NE system via Corticotropin-Releasing Factor (CRF) (Jedema et al., 2001; Valentino et al., 1983). We speculate that individuals exposed to psychosocial stress may amplify involuntary attentional resources for detecting potential social threats, such as new negative feedback, while diminishing voluntary attentional resources directed toward the cognitive task. Intriguingly, we noted a non-significant reduction in reaction times for correct responses among stressed subjects. This finding could signify that stress facilitated stimulus categorization, a top-down process (Connor et al., 2004). However, we believe that the decrease in reaction times relates to the sensitization and sensory discrimination processes that facilitate threat detection and enable rapid responses under stress conditions. In this context, compared to the control group, which exhibited a process of adaptation to the target stimulus, the stress group maintained the amplitude of N1 in both oddballs. Additionally, the amplitude of MMN increased after stress, indicating enhanced auditory sensitivity and discrimination (Näätänen et al., 2007). Based on our findings, we hypothesize that psychosocial stress augments bottom-up attentional resources while simultaneously reducing voluntary attentional resources devoted to the task. This effect could be accentuated in complex attentional tasks or daily activities that require more cognitive resources than the oddball paradigm.

On the other hand, a particular feature of our experimental design is that the co-investigator (a person who generates negative social evaluation) remained in the room during the second oddball (ODD POST). Suppose only the passive presence of the co-investigator can trigger a neuroendocrine response and attentional changes associated with the stress response. In that case, this could imply that psychosocial stress could rapidly generate allostatic overload in natural environments. However, our experimental design does not allow us to dissociate the effect of the acute psychosocial stress caused by the co-investigator in MITSEEG and his passive presence in the attentional task (ODD POST). New experimental designs are required to dissociate these effects.

### 4.3 Phasic pupillary responses

The primary variable influencing the pupillary diameter is the change in ambient luminosity (Clarke et al., 2003). However, several studies show that small changes in pupillary are associated with cognitive phenomena, including emotional states (Bradley et al., 2008), attention (Kang et al., 2014), and mental effort (van der Wel and van Steenbergen, 2018). These variations follow coherent changes in neural activity throughout the cortex regulated by arousal (Hou et al., 2005; Reimer et al., 2014). Evidence suggests that changes in pupillary dilation reflects fluctuations in LC's tonic and phasic active (Rajkowski et al., 2004). In this line, studies have shown that spontaneous and drug-induced drowsiness and other low-activation states, characterized by low tonic locus coeruleus activity, are accompanied by a decrease in basal pupil diameter (Hou et al., 2005; Morad et al., 2000). Conversely, noradrenergic drugs that increase activation and tonic locus coeruleus activity also increase basal pupil diameter (Phillips et al., 2000). Finally, a large number of studies have shown that task processing is accompanied by rapid and dramatic pupillary dilation (Beatty, 1982b, 1982a; Einhäuser et al., 2008; Richer and Beatty, 1987). Additionally, an fMRI study observed a positive relationship between the BOLD signal associated with phasic LC activity and pupil dilation induced by the auditory target stimulus in the oddball task (Murphy et al., 2014). These results are consistent with the emergence of a phasic locus coeruleus response to task-relevant events (Aston-Jones et al., 1994).

In our study, both groups auditory target stimulus elicited a phasic pupillary response, consistent with the occurrence of an LC phasic response to task-relevant events. However, stress group after MISTEEG showed decreased pupillary response associated with the target stimulus. We postulate that evoked pupillary response reduction reflects increased in LC tonic activity generated by MISTEEG. In this line, we observed a negative correlation between the physiological response to psychosocial stress (salivary cortisol and heart rate) and pupillary phasic activity (Fig. 10C and D). This result suggests that the L-HPA axis may modulate the mechanism underlying the relationship between pupillary diameter and LC activity.

In summary, we hypothesized and demonstrated that acute psychological stress decreased the phasic-evoked responses of the LC-NE system. The reduction of pupillary response to relevant stimulus (phasic response) after MISTEEG might reflect a hyper-alert state associated with increased tonic activity and phasic response inhibition of LC neurons.

### 4.4 Electrophysiological correlates of attention shift

In our study we observed a physiological and psychological response elicited by MISTEEG, subsequently accompanied by decreased performance in the attentional task. Following these results, we studied the components of the evoked potentials to evaluate the attentional effects of psychosocial stress in sensory processing.

The cluster-based exploratory analysis to study the effect of acute psychosocial stress on the electrodynamics of sensory processing showed a decrease in the evoked potential amplitude in stressed subjects. This effect emerged from 281 to 703 after the stimuli onset and was mainly localized to bilateral fronto-parietal sites (Fig. 6). Due to the spatio-temporal characteristics of the component, it probably represents brain activity dedicated to sensory processing or attentional control. However, unlike the ROI-based analysis, the cluster-based study found no significant differences in early time windows. This difference in results could be explained by the fact that large values of the cluster more heavily influence f-based cluster mass statistics. Given the nature of ERPs, this means they are generally more biased toward the peak of the effect (P3) (Fields and Kuperberg, 2020).

ERPs intra-subject ROI analysis showed that N1 decreased amplitude in ODD POST only in the control group (Fig. 7). As the target stimulus was physically identical in oddballs, N1 reduction could be explained in terms of the habituation process (Budd et al., 1998; Sokolov, 1963) and attentional effects (Bradley, 2009). Several studies show that N1 amplitude can reflect psychophysiological processes of habituation and orienting response (Budd et al., 1998; Fruhstorfer, 1971; Picton and Hillyard, 1976). Further, the amplitude of N1 to novel stimuli is linked to early attention processes and modulated by arousal (Näätänen, 2018; Rinne et al., 2006). In our study, the reduction of N1 in the control group may reflect a decrease in arousal due to the cognitive effort of MISTEEG. However, an alternative hypothesis is that the decline of N1 reflects an attenuated orientation response due to sensory habituation to the target stimulus. In this line, the repetition of the auditory oddball would cause sensory habituation.

Alternatively, within the stress group, we propose that the stable N1 amplitude observed across oddballs, coupled with the marked increase in MMN following MISTEEG, may indicate an enhancement of involuntary attention resources. This proposition aligns with the attentional gain theory. Intriguingly, stress impacts on N1 preservation are exclusively seen for the target stimulus. For the standard stimulus, however, both the control and stress groups display a diminished N1 amplitude during the second oddball (as illustrated in Fig. 7, S5). This finding implies that stress-related influences on the initial stages of processing predominantly correlate with the orientation response towards relevant or novel stimuli. In this sense, an emerging view on amygdala function considers it as a gatekeeper that assesses the environment for threat cues and facilitates enhanced sensory processing (leading to vigilance) by lowering perceptual thresholds in relevant sensory brain regions (Davis and Whalen, 2001). In acute stress states, vigilance is proposed to be primarily upregulated by fast-acting agents such as catecholamines, which increase cellular excitability in limbic areas, predominantly in the amygdala (de Kloet et al., 2005). A potential driving force of the stress effects might be found in the elevated activity of the LC-NE system (Nieuwenhuis et al., 2005). Given that the amygdala receives direct norepinephrinergic innervation from LC (Asan, 1998), and NE levels in the amygdala rise in response to stressful stimuli (Galvez et al., 1996). LC-NE system might promote a bottom-up attentional bias to potential stressors through this mechanism. We believe that subjects exposed to psychosocial stress increased their early attention processes associated with sensory processing via the LC-NE system. This effect which would explain the non-habituation to the target stimulus and the sustained or enhanced orientation response associated with N1 and MMN amplitude.

ERP intra-subject ROIs analysis shows that the stress group decreased P3s amplitude after MISTEEG in all ROIs (Fig. 9). In our oddball paradigm, attention-demanding stimuli elicit a P3a when the contents of working memory change. Working memory has been related to efficient top-down regulation of suppressing internal and external distractions (Arnsten, 2009). It could play a crucial role in the generation of rapid neural inhibition of ongoing activity to facilitate the transmission of stimulus/task information from frontal (P3a) to temporal-parietal (P3b) locations (Polich, 2007). Available data suggest that P3a is related to frontal focal attention and working memory mediated by dopaminergic activity (Polich, 2007). Interestingly, stress studies have observed high levels of catecholamines in periods of threat (Arnsten, 2009; Arnsten and Goldman-Rakic, 1998; Bloomfield et al., 2019), accompanied by a dramatic loss of prefrontal cognitive abilities (Arnsten and Goldman-Rakic, 1998; Qin et al., 2009). In this context, we interpret that the decrease of P3a in the stress group after MISTEEG reflect a sporadic impairment of prefrontal cortex functions, such as task-directed voluntary attentional control. This effect would be associated with losing an optimal balance between dopamine and norepinephrine levels in the frontoparietal cortex (Arnsten, 2009). In this sense, the evidence shows that psychosocial stress can impair functions associated with the prefrontal cortex, such as cognitive flexibility (Alexander et al., 2007), working memory (Luethi et al., 2009) and Top-Down attentional control (Palacios-García et al., 2021).

On the other hand, a reduction in amplitude in P3b could be related to a basal increase release of norepinephrine in the temporal-parietal cortex. P3b is associated with the temporal-parietal activity where dense norepinephrine inputs are found (Nieuwenhuis et al., 2005; Pineda, 1995; Pineda et al., 1989; Polich and Criado, 2006). A comprehensive review of the wide-ranging P3s neuropharmacology literature suggests that the LC-NE system underlies P3b generation for a target detection task (Nieuwenhuis et al., 2005). The suggestion that LC-NE contributes to P3 generation is consonant with attention resource allocation and human arousal-related effects (Intriligator and Polich, 1995; Kok, 2001). Functional neuroimaging studies support the idea that oddball responses depend on modulatory adrenergic inputs, mediated via β-adrenergic receptors (Strange and Dolan, 2007). In this context, the P3b response associated with to target stimulus reflects (at least in part) the neuromodulatory effect of phasic norepinephrine release in the neocortex (De Taeye et al., 2014; Nieuwenhuis, 2011; Nieuwenhuis et al., 2005). It is possible that a decrease in P3b amplitude post-exposure to acute psychosocial stress could be related to a high level of tonic activity of LC produced by social threat. The increased activity of the LC-NE system could affect the phasic release of noradrenaline in the temporal-parietal cortex that underlies the P3b response or generate desensitization of adrenergic receptors. These brain dynamics reflect a stereotypical stress response related to prioritizing threat exploration and fast responses, lowering neural network activity underlying top-down regulation.

**Fig. 11:**
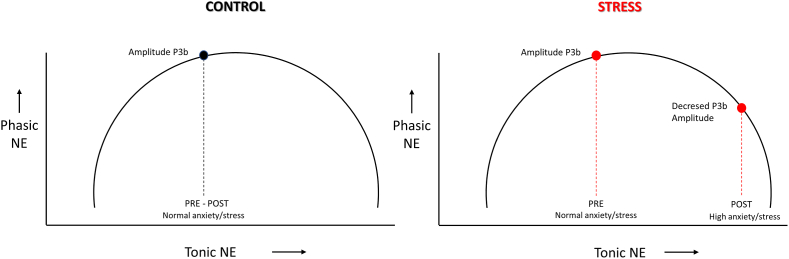
**Schematic of the effect of acute psychosocial stress on LC-NE system.** The diagram shows the effect of acute psychosocial stress on the potential mechanism of interaction between LC-NE activity, P3b amplitude and pupil phasic response to target auditory stimulus in the oddball auditory task. The level of tonic and phasic activity of the LC-NE system before and after MISTEEG is represented on the X and Y axes respectively. Inverted U curve describing the relationship between tonic noradrenergic activity, which can vary from low (inattentive states) to medium (alert conditions) to high (stress and other high-arousal states) and phasic noradrenergic activity, which is driven by a task-relevant stimulus.

In this sense, our results showed a positive relationship between the change in pupillary response and P3b (Fig. 10A). Further, with greater stress response during MISTEEG showed decreased P3b and pupillary response (Fig. 10B–D). Fig. 11 illustrates a descriptive model of the results observed in the oddball auditory task. The scheme shows the inverted U curve describing the relationship between tonic noradrenergic activity, which can vary from low (inattentive states) to medium (alert conditions) to high (stress and other high-arousal states) and phasic noradrenergic activity, which is driven by task-relevant (Nieuwenhuis et al., 2005). In the scheme, we incorporate the physiological effect of phasic norepinephrine release, reflected partly by the amplitude of P3b and pupillary phasic response. Acute psychosocial stress increases tonic LC-NE activity, which is associated with decreased pupillary phasic response and P3b amplitude. The inter-subject differences in P3b and pupil phasic response after MITSEEG reflect individual allostatic load of social threat.

### 4.5 Conclusions

Our results indicate that the acute stress protocol effectively generated physiological and subjective stress responses. This study shows that social threat affects the amplitude of ERPs components associated with orienting response (N1, MMN and P3a). These results could indicate that neural networks related to Bottom-up and Top-Down attentional control adopted different dynamics in periods of social threat. On the other hand, a decrease of P3b and the pupillary phasic response associated with cognitive tasks would be related to an increase in the tonic activity of the LC-NE system in situations of social threat. Under psychosocial stress experiences, we hypothesized that the operation of neural inhibition generated when cognitive mechanisms are engaged by stimulus and task demands are decreased.

### 4.6 Limitations

This study presents several limitations that should be acknowledged. Firstly, our research exclusively included male subjects in an attempt to control for gender-related variance in stress responses. However, as stress sensitivity fluctuates across the menstrual cycle, conducting comparable experiments involving both sexes in future research would be critical (Ossewaarde et al., 2010). Secondly, the participant demographic was limited to young to middle-aged individuals. Previous research suggests that age can modulate L-HPA reactivity (Seeman et al., 2001), thus extending the study to encompass a wider age range is vital.

Furthermore, our experimental design did not allow us to determine whether the stress effects on the orienting response, associated with N1, vary between task-relevant stimuli (target stimulus) and novel stimuli (non-target stimuli). Even though the P3 component has been linked with attention in the past, it could be viewed as a broader marker of cognitive processes, not exclusively reflecting attention. This makes the interpretation of the results nuanced, potentially necessitating exploration from both attentional and cognitive perspectives.

The study design also did not account for decreased alertness, and as such, the modulation of ERP components and pupillary response in a reduced tonic state of the LC-NE system was not investigated. Similarly, the EEG recording technique used did not facilitate a comprehensive analysis of signal source reconstruction. Future studies employing hemodynamic techniques could contribute to a more precise localization of the effects' sources observed in this research.

Finally, a significant challenge arose from the measurement of pupil diameter, revealing a notable study limitation. Our analysis demonstrated a marked difference in the statistical power to detect changes between the control and stress groups, the detection capacity being significantly diminished in the former. This discrepancy could have led to an imprecise comparison between the two groups, a limitation that future studies should seek to overcome.

## Funding

This work was supported by a PhD fellowship from CONICYT-PCHA/Doctorado Nacional/2016–21160904 to 10.13039/100005839GCA and the Fund for Innovation and Competitiveness (10.13039/501100016014FIC) of the Chilean Ministry of Economy, Development and Tourism, through the Millennium Science Initiative, Grant N°IS130005 and 10.13039/501100002848CONICYT/10.13039/501100002850FONDECYT 1150241.

## CRediT authorship contribution statement

**F. Rojas-Thomas:** Conceptualization, Methodology, Software, Formal analysis, Investigation, Data curation, Writing – original draft, Writing – review & editing, Visualization, Project administration. **C. Artigas:** Methodology, Investigation, Writing – review & editing. **G. Wainstein:** Formal analysis, Writing – original draft. **Juan-Pablo Morales:** Writing – original draft, Writing – review & editing. **M. Arriagada:** Formal analysis. **D. Soto:** Software. **A. Dagnino-Subiabre:** Conceptualization, Writing – review & editing, Funding acquisition. **J. Silva:** Funding acquisition. **V. Lopez:** Conceptualization, Writing – review & editing, Supervision, Funding acquisition.

## Declaration of competing interest

The authors report no potential financial conflicts of interest.

## Data Availability

Data will be made available on request.
