# Supplementary material for: Impact of acute psychosocial stress on attentional control in humans. A study of evoked potentials and pupillary response
Source: Neurobiol Stress. 2023 Jun 12;25:100551. doi: 10.1016/j.ynstr.2023.100551 (PMC10285563; doi:10.1016/j.ynstr.2023.100551)
Supplement: Multimedia component 1 [file mmc1.docx]

**ANEXXEED**

1. “Please concentrate.”
2. “Please try a little harder.”
3. “You are below average; please focus.”
4. “Your performance is not serving us for this experiment.”
5. “Your result will be invalidated for this experiment.”

**S1 Table. Phrases are used to generate a negative social evaluation.** Each sentence was used in order every 5 minutes in the arithmetic test for the stress group.

| **Effect: Main effect of time factor** | |
| --- | --- |
| **All Latencies show effects** | **Electrodes** |
| 258 - 703 ms | Fp1, AF7, AF3, F1, F3, F5, F7, FT7, FC5, FC3, FC1, C1, C3, C5, T7, TP7, CP5, CP3, CP1, P1, P3, P5, P7, P9, PO7, PO3, O1, Iz, Oz, POz, Pz, CPz, Fpz, Fp2, AF8, AF4, AFz, Fz, F2, F4, F6, F8, FT8, FC6, FC4, FC2, FCz, Cz, C2, C4, C6, T8, TP8, CP6, CP4, CP2, P2, P4, P6, P8, P10, PO8, PO4, O2 |
| 258 - 703 ms | C1, C3, C5, T7, TP7, CP5, CP3, CP1, P1, P3, P5, P7, P9, PO7, PO3, O1, Iz, Oz, POz, Pz, CPz, Fpz, Fp2, AF8, AF4, AFz, Fz, F2, F4, F6, F8, FT8, FC6, FC4, FC2, FCz, Cz, C2 |
| 266 - 703 ms | FC3, FC1, C1, C3, C5, T7, TP7, CP5, CP3, CP1, P1, P3, P5, P7, P9, PO7, PO3, O1, Iz, Oz, Poz, Pz, CPz, Fpz, Fp2, AF8, F4, AFz, Fz, F2, F4, F6, F8, FT8, FC6, FC4, FC2, FCz, Cz, C2, C4, C6, T8, TP8, CP6, CP4, CP2, P2, P4, P6, P8, P10, PO8, PO4, O2 |
| 539 - 703 ms | AF7, AF3, F1, F3, F5, F7, FT7, FC5, AF4, AFz, Fz, F2, F4, F6, F8, FT8, FC6, FC4 |
| 672 – 703 ms | Fp1, Fpz, Fp2, AF8. |
| **Effect: Interaction effect between group and time** | |
| 281 - 703 ms | Fp1, AF7, AF3, F1, F3, F5, F7, FT7, FC5, FC3, FC1, C1, C3, C5, T7, TP7, CP5, CP3, CP1, P1, P3, P5, P7, P9, PO7, PO3, O1, Iz, Oz, POz, Pz, CPz, Fpz, Fp2, AF8, AF4, AFz, Fz, F2, F4, F6, F8, FT8, FC6, FC4, FC2, FCz, Cz, C2, C4, C6, T8, TP8, CP6, CP4, CP2, P2, P4, P6, P8, P10, PO8, PO4, O2 |
| 281 - 703 ms | Iz, Oz, POz, PO8, O2 |
| 352 – 703 ms | C1, Oz, CPz, Cz, C2, C4, CP2, P2, P4, O2 |
| 359 – 703 ms | FC1, CP1, Pz, FC4, FC2, FCz, TP8, CP6, CP4, P6, P8, PO8, PO4 |
| 367 – 703 ms | F1, FC3, P1, Iz, POz, Fz, C6 |
| 375 - 703 ms | F3, F5, FC5, CP5, CP3, P3, AFz, F2, F4, F6, FC6 |
| 383 – 703 ms | Fp1, AF3, FT7, C5, T7, TP7, P5, P7, PO7, PO3, Fpz |
| 469 – 703 ms | F7, AF8, AF4 |
| 477 – 703 ms | F7, AF8, AF4 |
| 477 – 703 ms | AF7, P9 |

**S2 Table. Latencies and electrodes relative to factorial univariate test.** The table displays the electrodes included in the cluster and their respective latencies

| **Effect: Pair-wise comparisons between ODD PRE and ODD POST in both groups. A significant difference in ERP amplitude only in the stress group.** | |
| --- | --- |
| 266 – 703 ms | Fp1, AF7, AF3, F1, F3, F5, F7, FT7, FC5, FC3, FC1, C1, C3, C5, T7, TP7, CP5, CP3, CP1, P1, P3, P5, P7, P9, PO7, PO3, O1, Iz, Oz, POz, Pz, CPz, Fpz, Fp2, AF8, AF4, AFz, Fz, F2, F4, F6, F8, FT8, FC6, FC4, FC2, FCz, Cz, C2, C4, C6, T8, TP8, CP6, CP4, CP2, P2, P4, P6, P8, P10, PO8, PO4, O2 |
| 273 – 703 ms | P1, P3, Iz, Oz, POz, CPz, C2, CP4, CP2, P2, P4, PO4, O2 |
| 281 – 703 ms | O1, C4 |
| 289 – 703 ms | PO3 |
| 297 – 703 ms | P5, PO7 |
| 305 – 703 ms | C1, TP7, CP5, CP3, CP1, P7, Cz, C6 |
| 344 – 703 ms | FC1, C3, T7, FCz |
| 352 – 703 ms | FC5, FC3, Fz, FC2 |
| 359 – 703 ms | F1, F3, F5, FT7, T7, FC4 |
| 367 – 703 ms | AF3, AFz, F2, F4, F6, FC6, T8 |
| 375 – 703 ms | F7, AF8 |
| 383 – 703 ms | Fpz |
| 484 – 703 ms | AF4 |
| 531 – 703 ms | AF1, F8, FP2 |
| 539 – 703 ms | FP2 |

**S3 Table. Latencies and electrodes relative to group-by-time pair-wise comparisons factorial univariate test.** The table displays the electrodes included in the cluster and their respective latencies


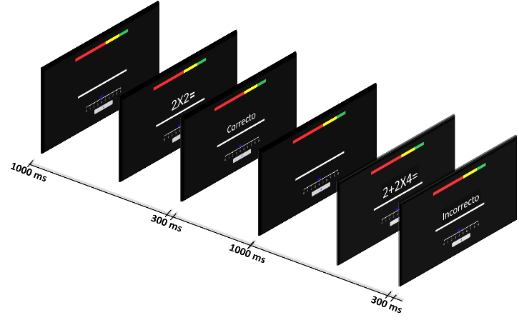

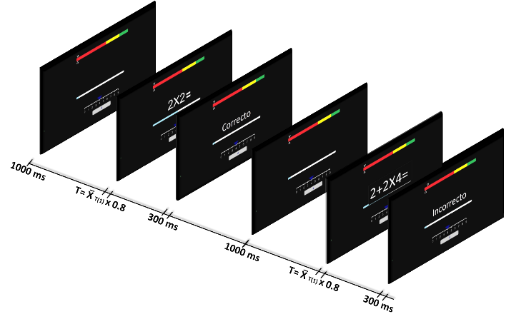

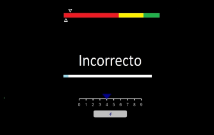

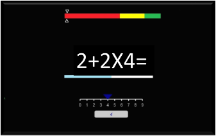
a)

b)


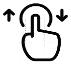


**S1 Fig. EEG-compatible modified version of the Montreal imaging stress task (MISTEEG).** (a) Control and stress version of MIST. (b) Response scheme, feedback and performance indicators in the stress version of MIST.


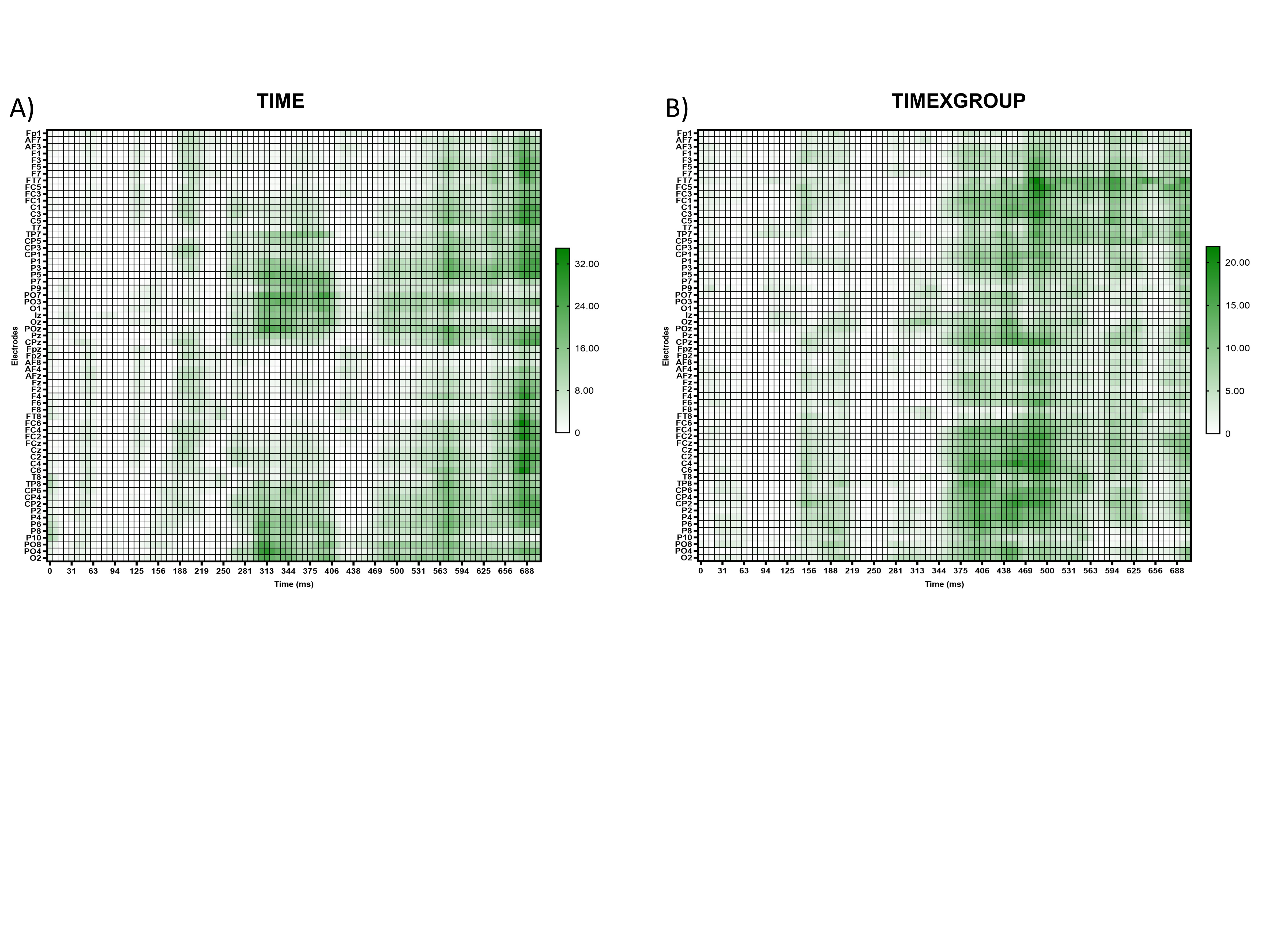


**S2 Fig. Cluster topography relative to the time factor and group-by-time interaction effect**. Raster diagram illustrating main effect for the factor time (A) and interaction between group and time (B) according to permutation test based on the cluster mass statistic. Each colored lectrode/timepoint represents a F value. Note that the electrodes are organized along the y-axis somewhat topographically. Electrodes on the left and right sides of the head are grouped on the figure’s top and bottom, respectively. Midline electrodes are shown in the middle. Within those three groupings, the y-axis top-to-bottom corresponds to the scalp anterior-to-posterior.


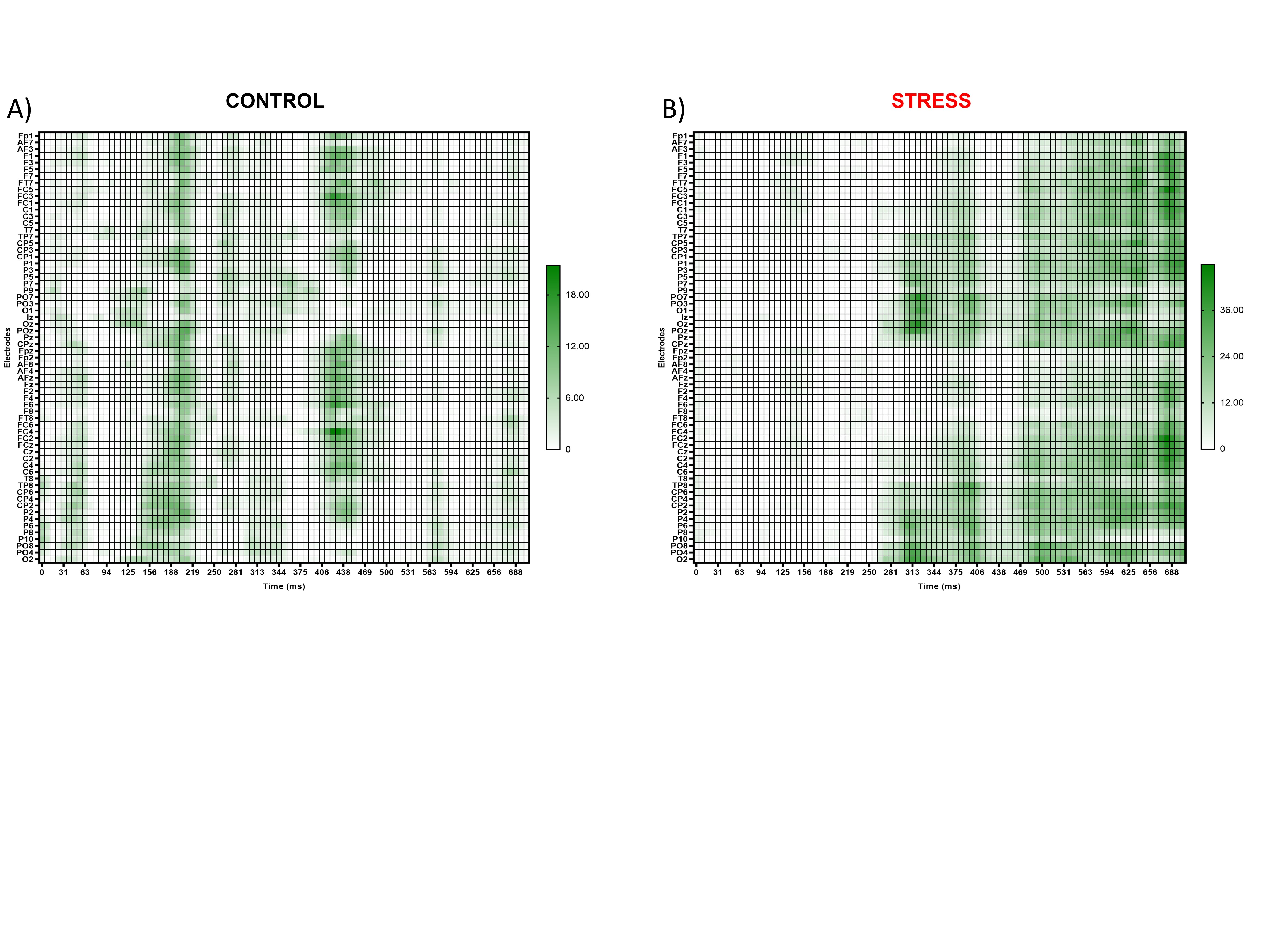


**S3 Fig. Cluster topography relative to group-by-time pair-wise comparisons**. Raster diagram of ERP amplitude differences between ODD PRE and ODD POST for control (A) and stress group (B) according to permutation test based on the cluster mass statistic. Each colored lectrode/timepoint represents a F value. Note that the electrodes are organized along the y-axis somewhat topographically. Electrodes on the left and right sides of the head are grouped on the figure’s top and bottom, respectively. Midline electrodes are shown in the middle. Within those three groupings, the y-axis top-to-bottom corresponds to the scalp anterior-to-posterior.


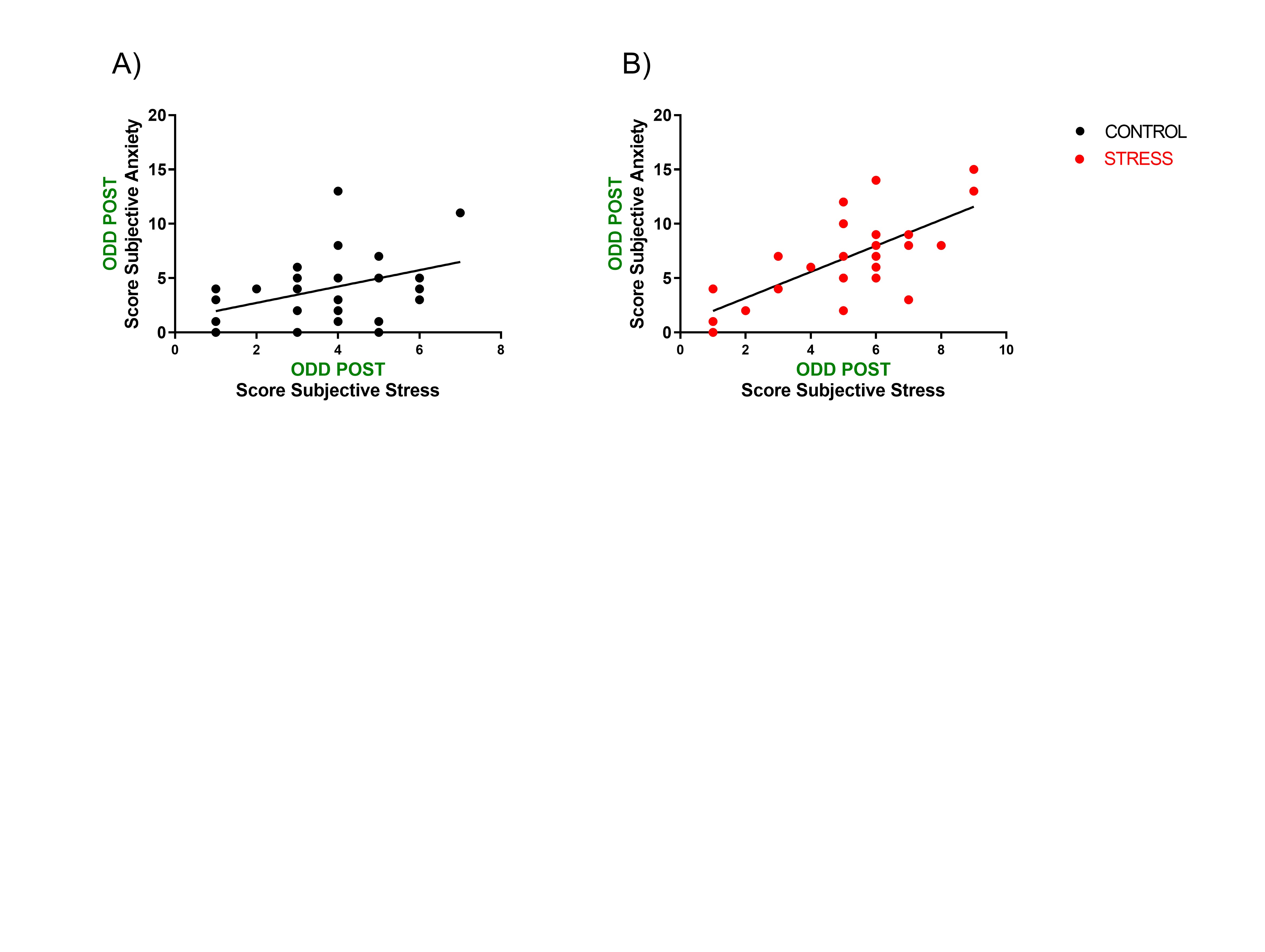


**S4 Fig.** **Spearman correlation between subjective stress and anxiety.** Association between stress and subjective anxiety experienced during the second auditory oddball (ODD POST) for the control group (A) and the stress group (B).


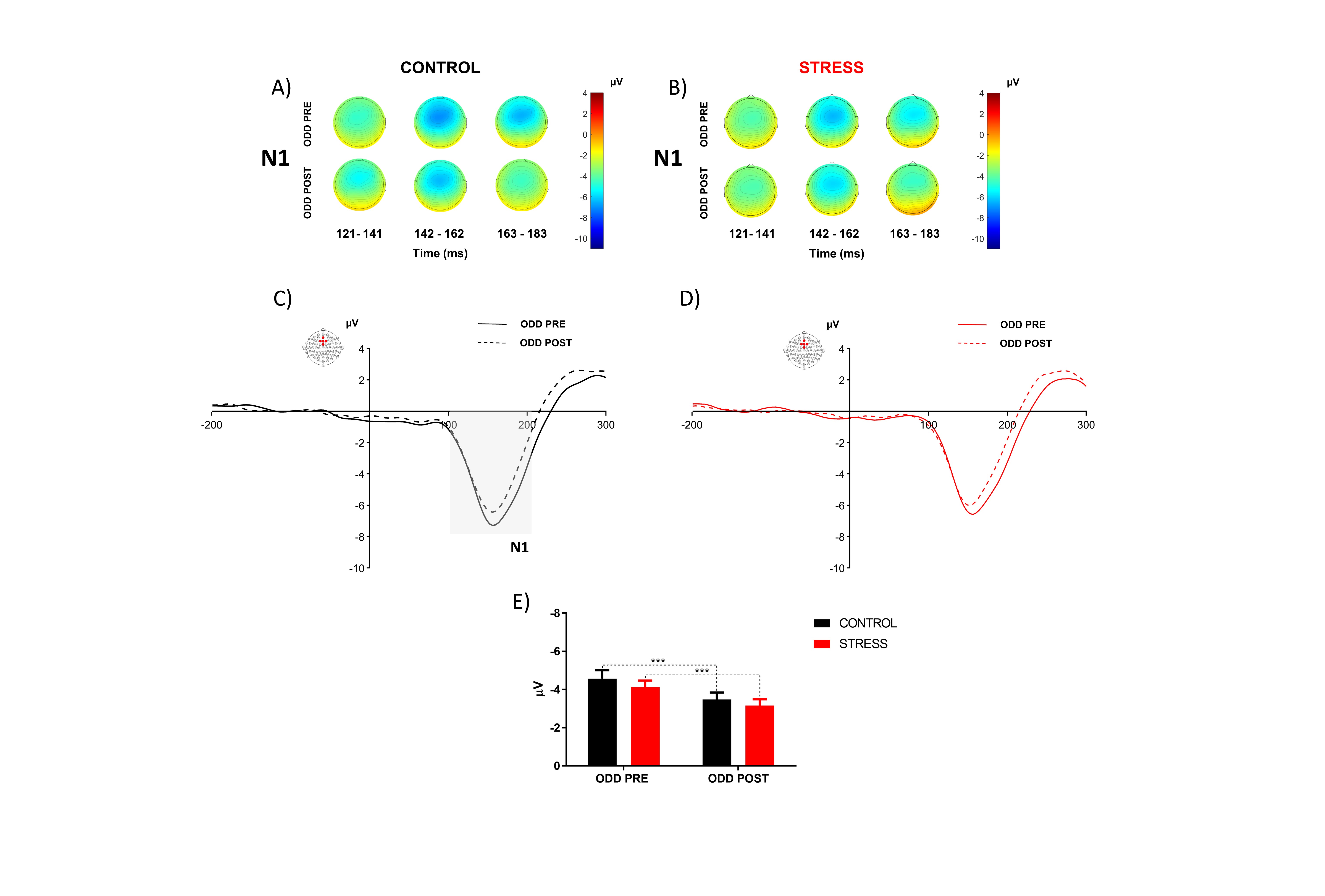


S5 Fig. **N1 amplitude associated with the standard stimulus.** Scalp distributions of N1 component associated with standard tone for ODD PRE and ODD POST (A, B). N1 component elicited to standard tone, the continuous line represented ODD PRE and the dashed line represented ODD POST (C, D). Average quantification of N1 amplitude for control and stress group (E). Error bars ± S.E.M. and asterisks indicate statistically significant differences (*: p<0.05).
